# Analytical quality-by-design approach for development and validation of HPLC method for the simultaneous estimation of omarigliptin, metformin, and ezetimibe: application to human plasma and dosage forms

**DOI:** 10.1186/s13065-023-00955-w

**Published:** 2023-05-05

**Authors:** Galal Magdy, Amira A. Al-enna, Fathalla Belal, Ramadan A. El-Domany, Ahmed M. Abdel-Megied

**Affiliations:** 1 Pharmaceutical Analytical Chemistry Department, Faculty of Pharmacy, Kafrelsheikh University, P.O. Box 33511, Kafrelsheikh, Egypt; 2 Pharmaceutical Analytical Chemistry Department, Faculty of Pharmacy, Mansoura University, P.O. Box 35516, Mansoura, Egypt; 3 Microbiology and Immunology Department, Faculty of Pharmacy, Kafrelsheikh University, P.O. Box 33511, Kafrelsheikh, Egypt; 4 Department of Pharmaceutical Sciences, School of Pharmacy, Notre Dame of Maryland University, Baltimore, MD 21210 USA

**Keywords:** Omarigliptin, Metformin, Ezetimibe, Quality by design, HPLC

## Abstract

A simple, selective, and sensitive RP-HPLC method was proposed for the simultaneous determination of two co-administered antidiabetic drugs (omarigliptin and metformin) with an anti-hyperlipidemic drug (ezetimibe) in a medicinally-recommended ratio of 2.5:50:1, respectively. The proposed procedure was optimized by adopting a quality-by-design approach. The influence of different factors on chromatographic responses was optimized by applying the two-level full factorial design (2^5^). The optimum chromatographic separation was achieved using Hypersil BDS C18 column at 45 °C, and the mobile phase pumped isocratically composed of methanol: potassium dihydrogen phosphate buffer (6.6 mM; pH 7, 67:33% *v/v*) at a flow rate of 0.814 mL/min using 235 nm as a detection wavelength. The developed method was capable of separating this novel mixture in less than 8 min. The calibration plots of omarigliptin, metformin, and ezetimibe showed acceptable linearity over the ranges of 0.2-2.0, 0.5–25.0, and 0.1-2.0 µg/mL with quantitation limits of 0.06, 0.50, and 0.06 µg/mL, respectively. The proposed method was successfully applied to determine the studied drugs in their commercial tablets with high % recoveries (96.8-102.92%) and low % RSD values (less than 2%). The applicability of the method was extended to the *in-vitro* assay of the drugs in spiked human plasma samples with high % recoveries (94.3-105.7%). The suggested method was validated in accordance with ICH guidelines.

**Supplementary Information:**

The online version contains supplementary material available at 10.1186/s13065-023-00955-w.

## Introduction

Type 2 diabetes mellitus occurs due to impaired insulin secretion from the β-pancreatic cells or due to insulin resistance in which cells don’t respond to the secreted insulin. Insulin resistance is commonly associated with obese patients. In response, the pancreatic β cells have to secrete high levels of insulin. However, functional defects in insulin secretion occur over time and then type 2 diabetes mellitus arises [1, 2]. For this reason, the master feature of successful therapy is to control endogenous insulin secretion by weight loss, exercise, diet, and some medications including oral hypoglycemics and anti-hyperlipidemics [1].

Omarigliptin (OMG) ((2*R*,3* S*,5*R*)-2-(2,5-difluorophenyl)-5-(2-methylsulfonyl-4,6-dihydropyrrolo[3,4-c]pyrazol-5-yl)oxan-3-amine) [3] (Additional file 1: Figure S1a) is a novel dipeptidyl peptidase 4 inhibitor which can be taken once weekly unlike other oral anti-diabetics which are usually taken once daily. It inhibits the inactivation of incretins and also motivates glucose dependent insulin release [4].

Metformin (MET) (3-(diaminomethylidene)-1,1-dimethylguanidine) [3] (Additional file 1: Figure S1b) is a biguanide oral hypoglycemic agent used in type 2 diabetes mellitus management. It improves tissues sensitivity to insulin and thus decreases the insulin resistance. It can be used either alone as an initial therapy or in combination with other drugs such as gliptins [2, 5].

Ezetimibe (EZT) ((3*R*,4* S*)-1-(4-fluorophenyl)-3-[(3* S*)-3-(4-fluorophenyl)-3-hydroxypropyl]-4-(4-hydroxyphenyl)azetidin-2-one) [3] (Additional file 1: Figure S1c) is considered the first anti-hyperlipidimic drug which prevents intestinal absorption of cholesterol from diet without affecting fat soluble nutrients absorption [6]. Furthermore, it helps to accomplish lipid treatment goals that need to be strictly enforced in patients with diabetes mellitus, so it seems to improve insulin resistance and that’s why it is co-administered with oral anti-diabetic drugs such as OMG and MET in management of non-insulin dependent diabetes mellitus [7].

The literature review showed that few reported methods were available for determination of OMG either alone or in combination with other drugs. These methods comprise HPLC [8–10], spectrofluorimetry [11], spectrophotometry [12], and LC-MS/MS [13]. Concerning MET, a number of reported methods was available including HPLC [14–20], spectrophotometry [21–24], spectrofluorimetry [25, 26], LC-MS/MS [27–30], and TLC [31, 32]. Different methods were also reported for the determination of EZT including HPLC [33–35], spectrophotometry [36–38], spectrofluorimetry [39], and LC-MS/MS [40].

To best of authors’ knowledge, no reported methods are yet available for the assay of OMG, MET, and EZT simultaneously. Accordingly, the aim of the present work was to design a rapid, simple, and sensitive RP-HPLC method for the analysis of the cited drugs in their commercial tablets and spiked human plasma simultaneously using quality by design (QbD) approach for the first time. As mentioned before, the ratio between the cited drugs is 2.5:50:1 for OMG, MET, and EZT, respectively as their recommended doses are 25 mg once weekly, 500 mg daily, and 10 mg daily for OMG, MET, and EZT, respectively [4, 41, 42], rendering their simultaneous analysis a challenging task. Therefore, development of QbD approach was crucial for the developed method to determine them simultaneously with high accuracy and sensitivity. The developed method was capable of separating this novel mixture in less than 8 min.

QbD is a statistics-dependent method that provides numerous benefits in the optimization of different analytical methods. It needs fewer efforts, time, and resources for optimization rather than the traditional ones. Additionally, setting up experimental design permits precise determination of the most important factors of the method. The two-level full factorial design (2^5^ FFD) was adopted in the current method; permitting the use of small number of experiments for studying large number of factors [43–47].

## Experimental

### Reagents and materials

OMG (99.0%) was obtained from the Center for Drug Research and Development (CARD, BUE), MET (99.7%) was provided by Amoun Pharmaceutical Co., Alobour City, Egypt, and EZT (100.0%, batch no. EM0080819) was obtained from National Organization of Drug Control and Research Center (NODCAR), Cairo, Egypt. Glucophage^®^ Tablets (500 mg MET/tablet, batch no. LIE 2986, Product of Minapharm Pharmaceutical Co., Cairo, Egypt), and Ezetimibe^®^ Tablets (10 mg EZT/tablet, batch no. 065007, Borg Pharmaceutical Co., Alexandria, Egypt) were attained from a local Pharmacy in the Egyptian market. Human plasma samples were obtained from Mansoura University Hospitals (Mansoura, Egypt) and maintained at -80 °C for further use after thawing. Phosphoric acid (99.0%), methanol, sodium hydroxide, and potassium dihydrogen phosphate were purchased from Sigma-Aldrich (St. Louis, MO, USA). Double distilled water was utilized during the study.

### Software and instrumentation

A Dionex UltiMate 3000 HPLC (Thermo Scientific, Dionex, CA, USA) equipped with a quaternary pump (LPG-3400SD), an auto-sampler (WPS-3000TSL), a diode array detector (DAD), and a column thermostat (TCC-3000SD) was utilized during the study. Hypersil BDS C18 column (250 mm × 4.6 mm id, 5 μm particle size) was also utilized. Software (Chromeleon 7) was used for acquirement and handling of data. Jenway 3510 pH-meter (UK), vortex mixer, IVM-300p (Gemmy Industrial Corp, Taiwan), and cooling centrifuge, 2–16KL (Germany) were also utilized. Minitab Release 16 Software (State College, USA) was used for the factorial design statistical analysis.

### Standard stock solutions

Stock solutions (100.0 µg/mL) of each of OMG, MET, and EZT were prepared by weighing 0.01 gm of each drug in a 100.0 mL volumetric flask and completing to the volume with methanol. Different concentrations were attained by appropriate dilution of stock solutions with filtered double distilled water. The prepared solutions were stable for a minimum of 10 days when kept at 4 °C.

### Chromatographic conditions

A reversed phase C18 column (250 mm × 4.6 mm id, 5 μm particle size) and a mobile phase composed of methanol: potassium dihydrogen phosphate buffer (6.6 mM; pH 7; 67:33% *v/v*) were used for separation of the ternary mixture. Isocratic elution was achieved at 0.814 mL/min flow rate with an injection volume of 20.0 µL at 45 °C. Column conditioning for 15 min before the analysis was carried out. The diode array detector was set at λ_max_ of 235 nm which was suitable for the determination of the minor components in the mixture.

### Procedures

#### Calibration curves

A group of laboratory-prepared mixtures of OMG, MET, and EZT were prepared by transferring different volumes from 100.0 µg/mL stock solutions into a set of 10 mL measuring flasks and then completing to the mark with distilled water to get serial concentrations ranging from 0.2–2.0, 0.5–25.0, and 0.1–2.0 µg/mL for OMG, MET, and EZT, respectively. 20.0 µL of each of these mixtures were injected three times at the optimum chromatographic conditions. Each drug concentration (µg/mL) was plotted versus the peak area to acquire the calibration curves and hence the corresponding regression equations.

#### Analysis of commercial tablets

As OMG (Omaril^®^) tablets are not available in the Egyptian market, they were prepared by mixing the drug in its pharmaceutical concentration with 15 mg of maize starch, 10 mg of magnesium stearate, 15 mg of lactose, and 20 mg of talc for each tablet. A precisely weighed amount of the powder equivalent to 25.0 mg OMG was transferred into a small flask, 50 mL of methanol were added, sonicated for 20 min, and then filtered into a 100 ml volumetric flask. The solution was diluted with methanol to the mark and mixed well. Accurately measured volumes from the filtrate were transferred into 10 mL volumetric flasks and the procedure of analysis was completed as described under Section “Calibration curves”. The corresponding regression equations were used to compute the content of tablets.

10 tablets of each of Glucophage^®^ and Ezetimibe^®^ were individually weighed and homogenously ground. A precisely weighed amount of the powder equivalent to 500.0 mg of MET or 10.0 mg of EZT was transferred into a small flask, then complete the steps as mentioned above.

#### Spiked human plasma samples

1 mL aliquots of human plasma were transferred individually into a group of 15 mL centrifugation tubes. Serial concentrations ranging from 0.2–0.3, 1.0–5.0, and 0.1–0.3 µg/mL of OMG, MET, and EZT, respectively were added to the plasma samples then vortex mixed for 90 s. Methanol was added up to 4 mL for protein precipitation then, the tubes were centrifuged for 15 min at 6000 rpm. Filtration of each tube supernatant was carried out by syringe filters (0.45 μm). Determination of each drug concentration was carried out as described under Section “Calibration curves”. The concentration of the drugs in plasma was obtained using the corresponding regression equations.

All methods were performed in accordance with relevant guidelines and regulations and all experimental protocols were approved by the Committee of Research Ethics in the Faculty of Pharmacy, Kafrelsheikh University, Kafrelsheikh, Egypt.

#### Experimental design

QbD is a set of experiments used to simultaneously evaluate a group of factors at a definite number of levels by the least number of experiments to determine the optimum conditions. In this work, 2^5^ full factorial design, response optimizer, and optimization plots were used. 2^5^ FFD was carried out by 32 designed experiments to examine the optimum conditions that result in the best response values [46, 48] as shown in Additional file 1: Table S1.

**Fig. 1:**
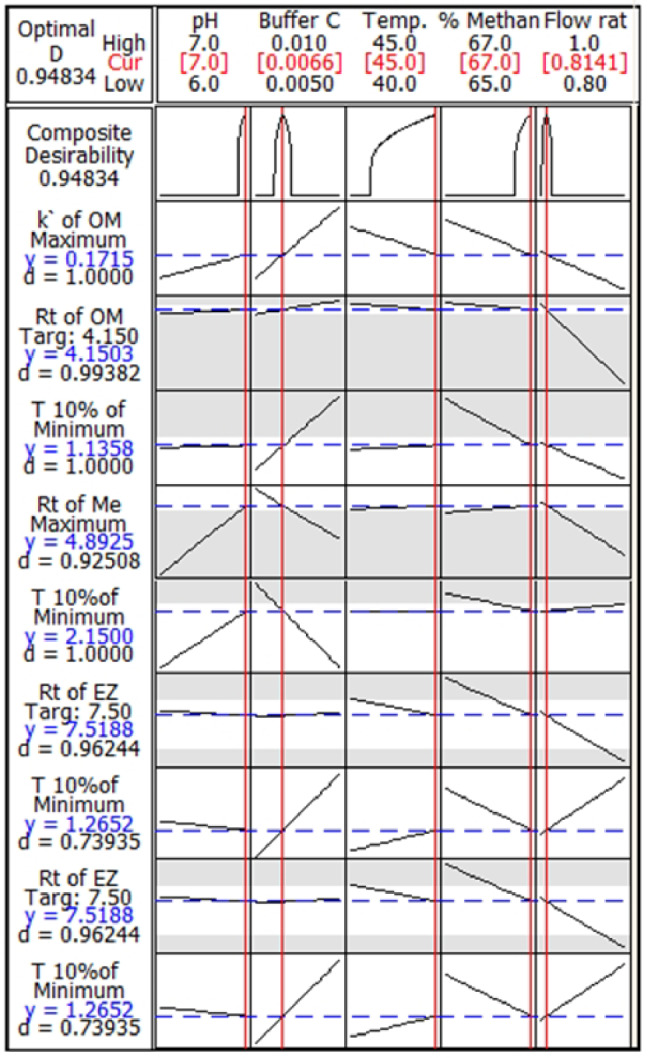
2^5^ Full factorial design (FFD) optimization plot

Concerning response optimizer, three values (upper, lower, and target) should be determined for each response. The optimum setting for the input variables plus desirability value that ranges from 0 to 1 and divided into composite desirability (D) and individual desirability (d) can be determined by Minitab Response Optimizer. The composite desirability should be maximized and the important values will be in the range of 0.1–10. All of the responses are equally significant except for T_10%_ of MET, therefore, the default value of 1 was given to each response except for T_10%_ of MET which was given the value of 2 (Table 1). The optimization plot, drawn by Minitab, also shows the impact of each factor on responses (Fig. 1) [43, 46].

**Table 1:** Response optimization of 2^5^ full factorial design for RP-HPLC separation of OMG/MET/EZT mixture

Parameters								
	Goal	Lower	Target	Upper	Weight	Import	Predicted Responses	Individual Desirability (d)
*K*‘_OMG_	Maximize	0.15	0.16	-	1	1	0.1715	1.0
Rt_OMG_	Target	4.1	4.15	4.2	1	1	4.15	0.9938
T_10%OMG_	Minimize	-	1.15	1.18	1	1	1.1358	1.0
Rt_MET_	Maximize	4.8	4.9	-	1	1	4.892	0.9250
T_10%MET_	Minimize	-	2.15	2.25	1	2	2.15	1.0
Rt_EZT_	Target	6.5	7.5	8	1	1	7.518	0.9624
T_10%EZT_	Minimize	-	1.2	1.45	1	1	1.265	0.7393
Optimum conditions: Organic modifier % = 67%, buffer conc.= 6.6 mM, pH = 7.0, temperature = 45 °C, flow rate = 0.814 mL/min							Composite desirability (D) = 0.9483	

## Results and discussion

This study aimed to develop a simple and rapid methodology for the concurrent estimation of OMG, MET, and EZT by RP-HPLC-DAD method for the first time using 2^5^ full factorial design. Well resolved and separated peaks for the studied drugs were achieved in less than 8 min. Preliminary experiments showed that organic modifier type and ratio, concentration of phosphate buffer, pH, temperature, and flow rate were the most important factors to be studied to determine the most optimum chromatographic conditions.

### Method development and optimization

The absorption spectra of the studied drugs were recorded and it was concluded that setting the wavelength of the DAD at 235 nm achieved the optimal sensitivity for all of the studied drugs.

C18 Hypersil BDS column (250 mm × 4.6 mm id, 5 μm particle size) and Chromolith WP 300 Epoxy (100 mm ×4.6 mm, Hr. Peters, Merck KGaA) monolithic column were tested. The experiments showed that C18 column produced high resolution and symmetrical peaks, while the monolithic column couldn’t produce suitable resolution.

pH of mobile phase had a great effect on retention time (Rt), peak area, and peak shape of the studied drugs because of its effect on the ionization of these drugs. OMG, MET, and EZT are basic in nature and the values of their log p were 0.1, -2.6, and 4.5, respectively [3]. Thus, they were delayed when pH increased due to suppression of their ionization. Therefore, pH values of the buffer solutions from 6 to 7 and different ratios of methanol from 65 to 67% were tested in accordance with the experimental design (Additional file 1: Table S1).

The organic solvent type was tested, and methanol was found to be the best one in concerning the symmetry of the peaks and resolution of studied drugs in less than 8 min. On the other hand, acetonitrile produced overlapped peaks and extended the time of analysis.

#### Experimental design

Some preliminary experiments were done prior to carrying out the experimental design to check its applicability. Two experimental sets for each factor were performed to determine the suitable domain for each one [46, 49] (Additional file 1: Table S1).

In order to obtain the most optimum chromatographic conditions, the most significant factors were introduced into Minitab 16. Selection of the factor levels was cautiously done to obtain an experimental domain that achieved the experimental qualifications. The preliminary experiments showed that % methanol (65.0–67.0% *v/v*), pH (6–7), buffer concentration (5–10 mM), temperature setting (40–45 °C), and flow rate (0.8-1.0 mL/min) were the optimal ranges in the 2^5^ FFD which recommended a group of thirty two experimentations indicating impacts of the stated factors on chromatographic responses (Additional file 1: Table S1).

##### Response optimization

This stage is considered as a compromise amongst numerous responses. In this step, we determine the lower, upper, and target values for Rt, capacity factor (k’), and T_10%_ (OMG), Rt and T_10%_ (MET), and Rt, T_10%_ (EZT). The Minitab Response Optimizer software was used for the determination of the optimal settings of input variables and then, desirability values. In accordance with optimization plot (Fig. 1) and the response optimizer, it was confirmed that, 67% *v/v* methanol, buffer concentration of 6.6 mM and pH value of 7 with column temperature of 45 °C and flow rate of 0.814 mL/min were the optimum chromatographic conditions (Table 1).

As shown in Pareto Charts and the main effect plots (Additional file 1: Figures S2 and S3), it was concluded that, flow rate was the most significant parameter affecting Rt of OMG and EZT, while pH was the most significant parameter affecting Rt and T_10%_ of MET. Buffer concentration had the largest effect on T_10%_ of OMG and EZT. In addition, % *v/v* methanol had the highest effect on k’ of OMG. All of these parameters have statistically significant effects at alpha = 0.05, except for the effect of buffer concentration on T_10%_ of OMG (Additional file 1: Figure S4).

### Method characteristics

**Fig. 2:**
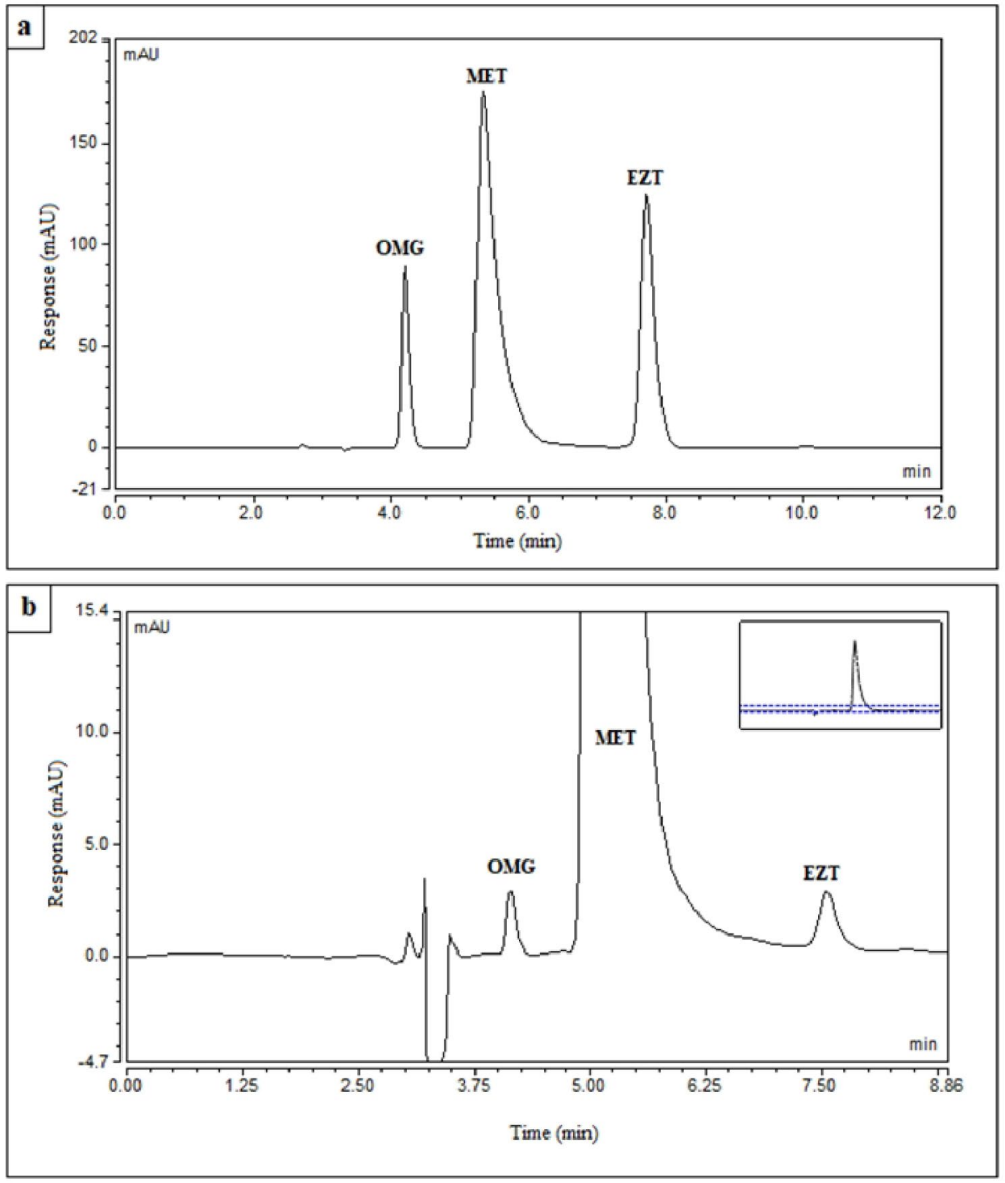
Typical chromatograms of **(a)** Mixture of OMG, MET, and EZT (25.0 µg/mL of each one) and **(b)** Mixture of OMG (0.5 µg/mL), MET (10.0 µg/mL), and EZT (0.2 µg/mL) at the optimum chromatographic conditions

A mixture containing equal concentrations of each of OMG, MET, and EZT was prepared for the selection of the most significant factors affecting the developed method. Then, 2^5^ full factorial design was utilized and the results were saved on computer. The attained chromatograms were analyzed for determination of the most important factors (Additional file 1: Table S1). The chromatograms of OMG/MET/EZT mixture at the optimal chromatographic conditions and system suitability parameters of the suggested procedure are presented in Fig. 2 and Additional file 1: Table S2, respectively.

### Method validation

The developed procedure was validated in accordance with ICHQ2 (R1) guidelines [50].

#### Linearity and range

The concentrations of each drug (µg/mL) were plotted versus their peak areas (P) to construct the calibration curves. The linear ranges were found to be 0.2-2.0, 0.5–25.0, and 0.1-2.0 µg/mL for OMG, MET, and EZT, respectively (Table 2). The corresponding regression equations that represent linear relationships are as follows:

1 $$P = 0.43\,C + 5 \times {10^{ - 5}}\,\,\,\,\,for\,OMG$$

2 $$P = 2.46\,C - 0.23\,\,\,\,\,for\,MET$$

3 $$P = 0.85\,C + 0.11\,\,\,\,\,for\,EZT$$

Where, P is the peak area and C refers to cited drugs concentration in µg/mL. Correlation coefficients (r) values were close to 1 indicating the good linearity of the proposed method.

**Table 2:** Analytical performance data for the proposed Method

Parameter	OMG	MET	EZT
Linearity range (µg/mL)	0.2-2.0	0.5–25.0	0.1-2.0
Limit of detection (LOD)^a^(µg/mL)	0.02	0.18	0.02
Limit of Quantitation (LOQ)^b^(µg/mL)	0.06	0.50	0.06
Regression equation	P = 0.43 C + 5 × 10^− 5^	P = 2.46 C – 0.23	P = 0.85 C + 0.11
Correlation coefficient (r)	0.9999	0.9999	0.9999
Standard deviation (S.D)	1.97	1.36	1.83
Percentage relative standard deviation (%RSD)	1.97	1.36	1.83
S.D. of the residuals (S_y/x_)	0.004	0.25	0.009
S.D. of the intercept (S_a_)	0.002	0.14	0.005
S.D. of the slope (S_b_)	0.002	0.01	0.005

^a^ LOD = 3.3 S_a_/b, ^b^ LOQ = 10 S_a_/b, where S_a_ = standard deviation of the intercept and b = slope.

#### Limits of quantitation (LOQ) and detection (LOD)

LOQ and LOD values were determined and their values were abridged in Table 2. The obtained results revealed the high sensitivity of the suggested procedure, which permitted its use for the estimation of the cited drugs in human plasma samples.

#### Accuracy and precision

The accuracy of the suggested procedure was estimated using the mean recovery percentages of the studied drugs in raw materials (Table 3) and five laboratory-prepared mixtures in a ratio of 2.5:50:1 for OMG, MET, and EZT, respectively (Additional file 1: Table S3). High values of percentage recoveries (96.8-102.82%, 97.2-101.21%, and 97.67-104.15%) for OMG, MET, and EZT, respectively were attained verifying the acceptable accuracy of the developed procedure.

**Table 3:** Application of the proposed method for the determination of OMG, MET, and EZT in pure forms

Parameter	OMG		MET		EZT	
	Conc. taken (µg/mL)	%Found^a^	Conc. taken (µg/mL)	%Found^a^	Conc. taken (µg/mL)	%Found^a^
	0.2	97.5	0.5	97.2	0.1	100.7
	0.25	100.44	1.0	99.54	0.2	104.15
	0.3	96.8	2.5	98.2	0.3	100.2
	0.5	102.82	5.0	101.21	0.4	99.2
	0.75	100.97	10.0	99.83	0.5	100.52
	1.0	98.83	15.0	100.97	1.0	97.67
	1.5	100.79	20.0	99.06	1.5	100.93
	2.0	99.65	25.0	100.25	2.0	100.01
Mean		99.73		99.53		100.42
± SD		1.97		1.36		1.83
%RSD		1.972		1.365		1.825
% Error		0.695		0.48		0.648

^a^ Mean of three determinations.

To estimate the inter-day and intra-day precisions, three different concentrations of the studied drugs and three replicates of each concentration were utilized. The obtained results showed low % RSD values (less than 2%) proving good precision of the suggested method (Additional file 1: Tables S3 and S4).

#### Robustness

The method’s robustness was investigated by checking the impact of minor variations in the experimental parameters on chromatographic responses. The effect of % methanol (67% ± 1%), buffer strength (6.6 mM ± 0.1), pH (7 ± 0.1), flow rate (0.814 ± 0.1), and temperature (45 °C ± 1 °C) were examined and showed non-significant effect on % RSD values and % recoveries (Additional file 1: Table S5).

#### Method selectivity

The selectivity of proposed method was evaluated by its ability to determine the studied drugs in raw material (Table 3) and prepared mixtures covering the linearity ranges (Additional file 1: Table S3). The results showed low %RSD and high % recoveries. Additionally, the method selectivity was proved by its capability to estimate the drugs in their dosage forms (Table 4) with high % recoveries and low % RSD values (less than 2%) without any interfering peaks, demonstrating that there was no interference from excipients at the optimal conditions. The selectivity of the method was also verified by analysis of studied drugs in spiked human plasma samples (Additional file 1: Tables S6 and S7). The developed method had the ability to determine the studied drugs in complex biological matrices with low % RSD values and high % recoveries (95.5-103.44%, 94.32-104.79%, and 94.3-105.7% for OMG, MET, and EZT, respectively) indicating that the matrix had a negligible effect on their retention times. Moreover, the selectivity of the method was confirmed by the high resolution factors among the peaks of studied analytes (Fig. 2).

**Table 4:** Application of the proposed method for the determination of OMG, MET and EZT in tablets

Drug	OMG		MET		EZT	
Pharmaceutical preparation	Prepared Omaril® tablets (25 mg OMG/tab)		Glucophage® tablets (500 mg MET /tab)		Ezetimibe® tablets (10 mg EZT/tab)	
	Conc. taken (µg/mL)	%Found ^a^	Conc. taken (µg/mL)	%Found ^a^	Conc. taken (µg/mL)	%Found ^a^
	0.20	99.75	0.5	101.06	0.10	99.6
	0.25	98.56	1.0	98.41	0.20	96.80
	0.30	100.87	2.5	102.26	0.30	97.33
	0.40	99.78	5.0	98.06	0.40	99.63
	0.50	99.36	10.0	101.34	0.50	102.92
	1.0	99.64	15.0	98.46	1.0	100.71
	1.5	101.77	20.0	101.21	1.5	99.91
	2.0	99.15	25.0	99.62	2.0	99.80
Mean ± SD		99.86 ± 1.01		100.05 ± 1.62		99.59 ± 1.9

^a^ Mean of three separate determinations.

#### System suitability

High performance of the proposed method was verified by the low % RSD values of the system suitability parameters obtained experimentally. The system suitability parameters were obtained experimentally and also from equations from 2^5^ FFD (Table S2). The values obtained in both cases were almost close to each other confirming that FFD can be used as a substitute for assessment of system suitability factors saving time, efforts, and resources [46].

### Applications

#### Analysis of the studied drugs in their tablets

The proposed method was applied for the determination of the studied drugs in their commercial tablets (prepared Omaril^®^, Glucophage^®^, and Ezetimibe^®^) without interference from the common tablet excipients (Table 4). The obtained mean % recoveries were found to be 99.86 ± 1.01, 100.05 ± 1.62, and 99.59 ± 1.9 for OMG, MET, and EZT, respectively with small values of % RSD values confirming the potential for effective use of the developed procedure for analysis of the cited drugs in their tablets.

#### Application to spiked human plasma samples

The studied drugs were simultaneously analyzed in spiked human plasma according to their therapeutic levels. The maximum plasma concentration (C_max_) of OMG was stated to be 0.3 µg/mL within 1 h after an oral dose of 25 mg/day [51]. Maximum MET plasma levels doesn’t exceed 5 µg/mL even at maximum doses [52], while C_max_ of EZT was reported to be 0.004 µg/mL after taking a dose of 10 mg daily [53]. The proposed method sensitivity was down to 0.2 and 0.5 µg/mL for OMG and MET, respectively which permitted its successful use for the determination of the two drugs in spiked plasma, while for EZT, this method can be useful for its determination in case of toxicity with over doses.

Applying the developed approach, linear relationships were achieved in spiked plasma samples with good correlation coefficients. The regression equations that represent linear relationships are as follows:

4 $$P = 1.3941\,C - 0.1884\,\,\,\,\,(r = 0.9797)\,\,\,\,\,for\,OMG$$

5 $$P = 3.1266\,C - 0.6541\,\,\,\,\,(r = 0.9971)\,\,\,\,\,for\,MET$$

6 $$P = 0.7625\,C - 0.077\,\,\,\,\,(r = 0.9952)\,\,\,\,\,for\,EZT$$

Where, P is the peak area, C refers to drug concentration (µg/mL) and r represents the correlation coefficients.

**Fig. 3:**
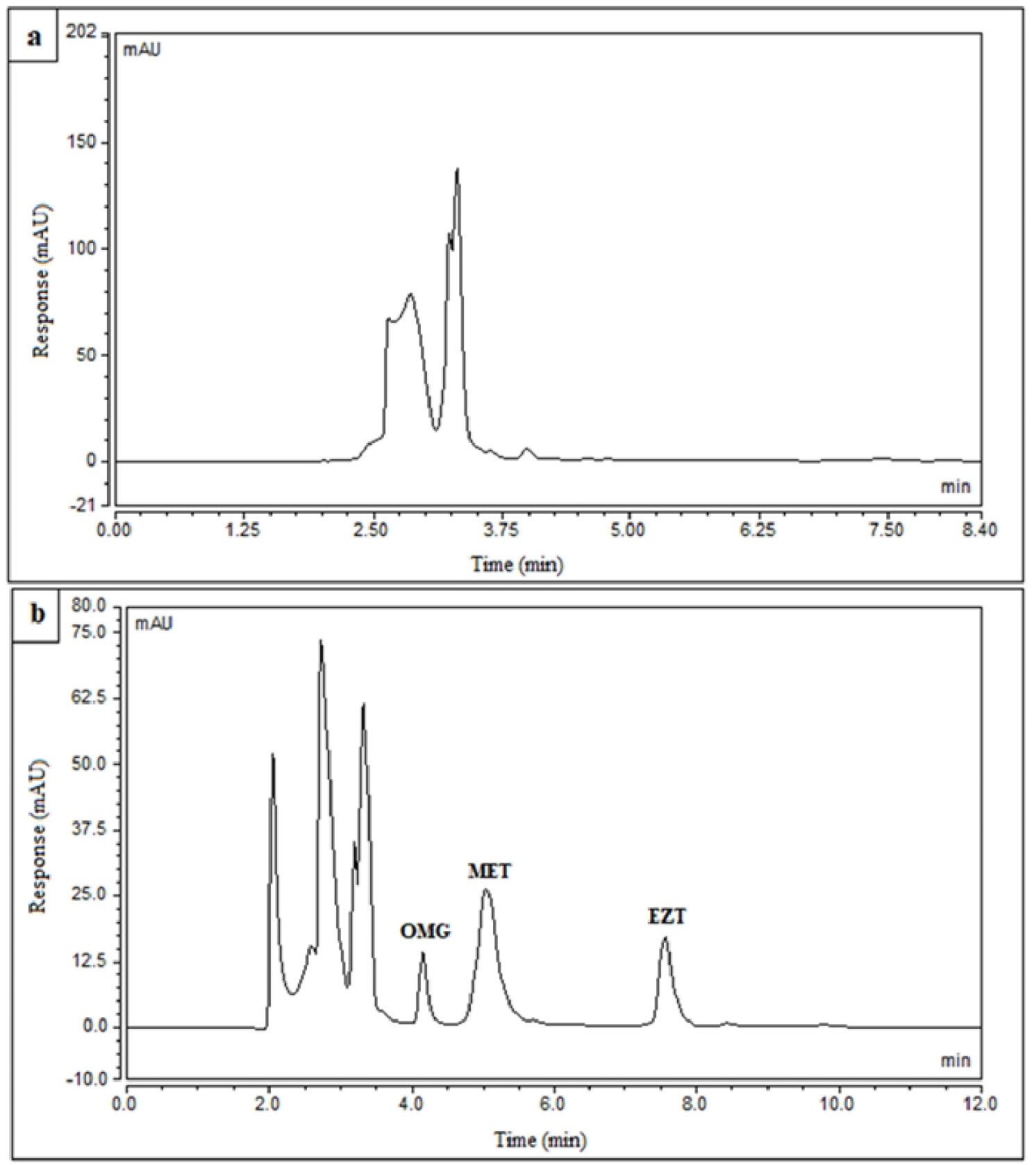
Typical chromatograms of **(a)** Blank plasma, **(b)** Mixture of OMG, MET, and EZT (2.0 µg/mL of each one) in spiked human plasma

Concerning the matrix effect, the proposed approach showed a good selectivity towards the studied drugs in spiked plasma with acceptable values of % recoveries in the range of (94.3-105.7%) for the three drugs and low % RSD values confirming that there was no interference from endogenous plasma components on Rts of the drugs as presented in Additional file 1: Tables S6 and S7 and Fig. 3.

## Conclusion

This work represents the first RP-HPLC method for the simultaneous estimation of OMG, MET, and EZT in a ratio of 2.5:50:1, respectively in less than 8 min. Optimization of the method was carried out using 2^5^ FFD. Owing to the high selectivity and sensitivity of the suggested method, the studied drugs could be estimated in spiked human plasma samples without interference and with acceptable values of % recoveries. Moreover, the proposed approach was utilized for analysis of studied drugs in their dosage forms with acceptable recovery results. ICH recommendations were used for method validation.

## Electronic supplementary material

Below is the link to the electronic supplementary material.

Supplementary Material 1

## Data Availability

The datasets generated and/or analyzed during the current study are available from the corresponding author on reasonable request.
