# Supplementary material for: Analytical quality-by-design approach for development and validation of HPLC method for the simultaneous estimation of omarigliptin, metformin, and ezetimibe: application to human plasma and dosage forms
Source: BMC Chem. 2023 May 5;17(1):45. doi: 10.1186/s13065-023-00955-w (PMC10163694; doi:10.1186/s13065-023-00955-w)
Supplement: Supplementary file 1 — Supplementary Material 1 [file 13065_2023_955_MOESM1_ESM.pdf]

Supplementary Information

Analytical quality-by-design approach for development and validation of HPLC method for the simultaneous estimation of omarigliptin, metformin, and ezetimibe: Application to human plasma and dosage forms

Galal Magdy [1*], Amira A. Al-enna [1], Fathalla Belal [2], Ramadan A. El-Domany [3], Ahmed M. Abdel-Megied [1,4]

[1] Pharmaceutical Analytical Chemistry Department, Faculty of Pharmacy, Kafrelsheikh University, Kafrelsheikh, P.O. Box 33511, Egypt

[2] Pharmaceutical Analytical Chemistry Department, Faculty of Pharmacy, Mansoura University, Mansoura, P.O. Box 35516, Egypt

[3] Microbiology and Immunology Department, Faculty of Pharmacy, Kafrelsheikh University, Kafrelsheikh, P.O. Box 33511, Egypt

[4] Department of Pharmaceutical Sciences, Notre Dame of Maryland University, School of Pharmacy, Baltimore, MD 21210, USA

***Corresponding author:** Galal Magdy

E-mail address: galal_magdy@pharm.kfs.edu.eg

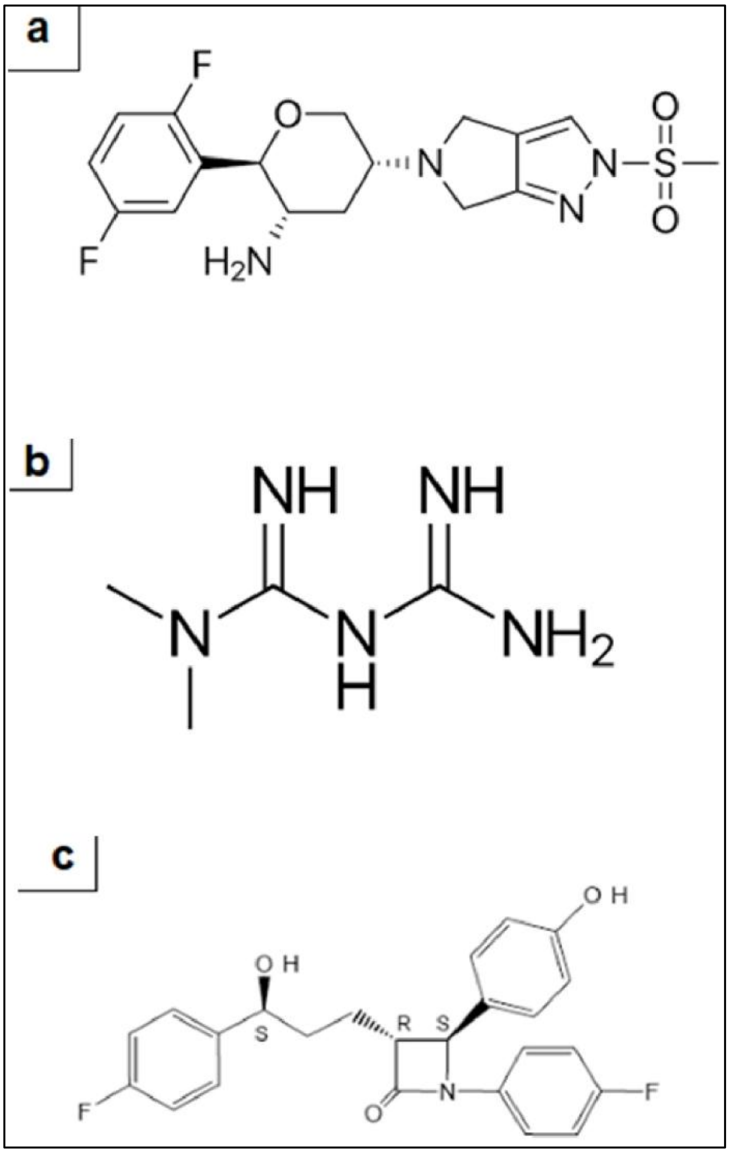

Fig. S1 Structural formulae of OMG (a), MET (b), and EZT (c).

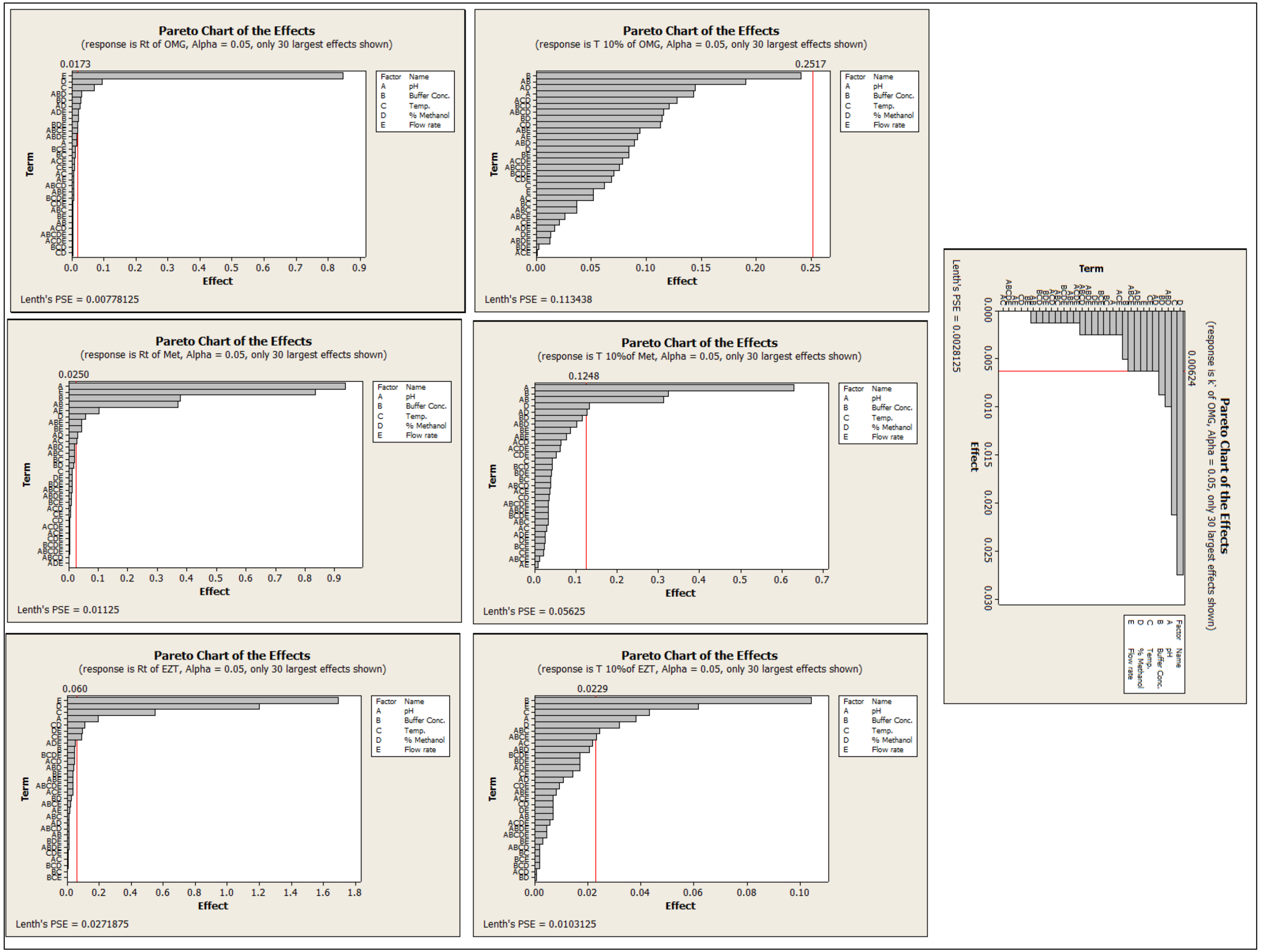

Fig. S2 2^5 FFD Pareto charts of the effects on the chromatographic responses at alpha = 0.05.

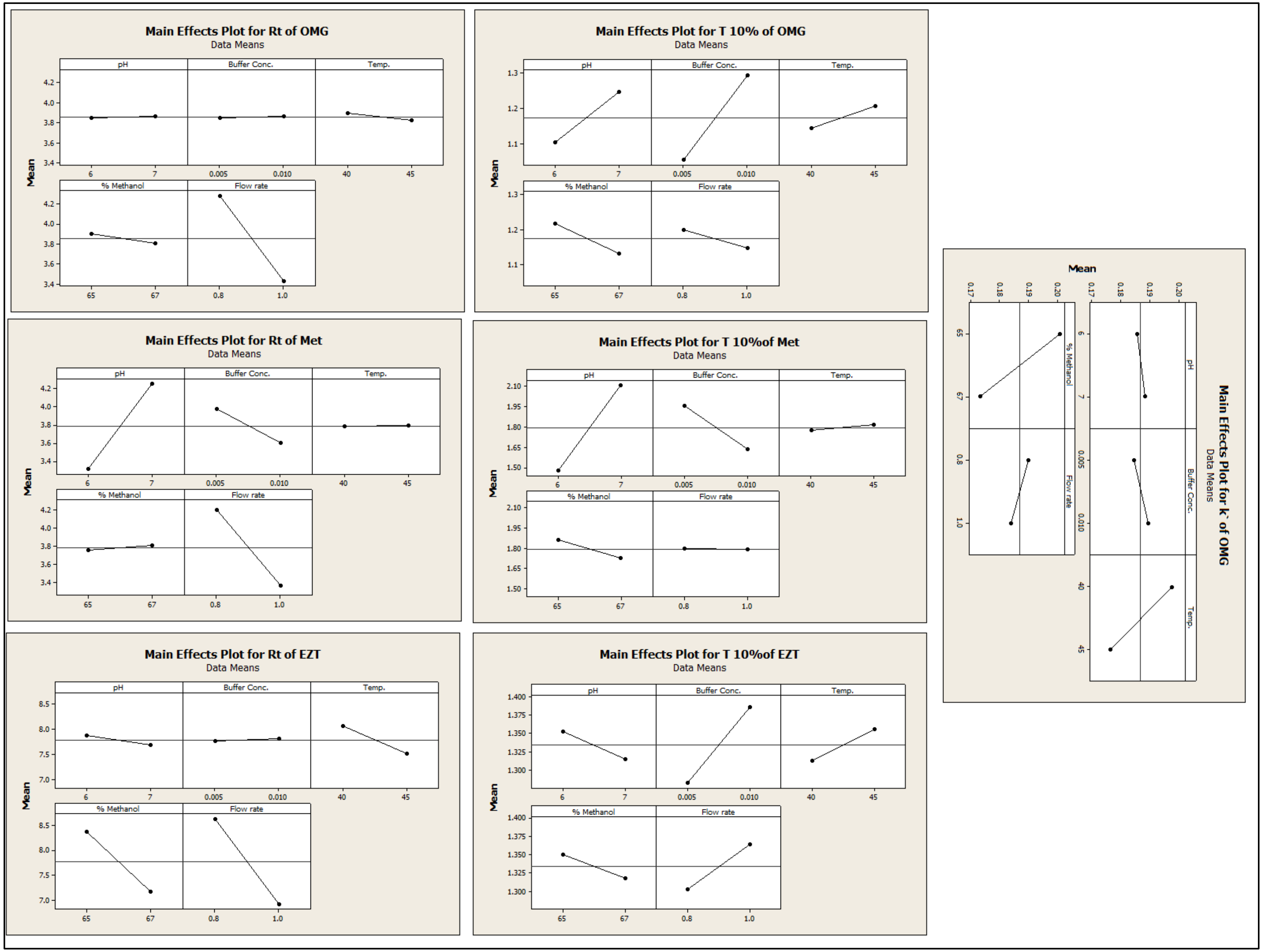

Fig. S3 2^5 FFD main effect plots for chromatographic responses by data means type.

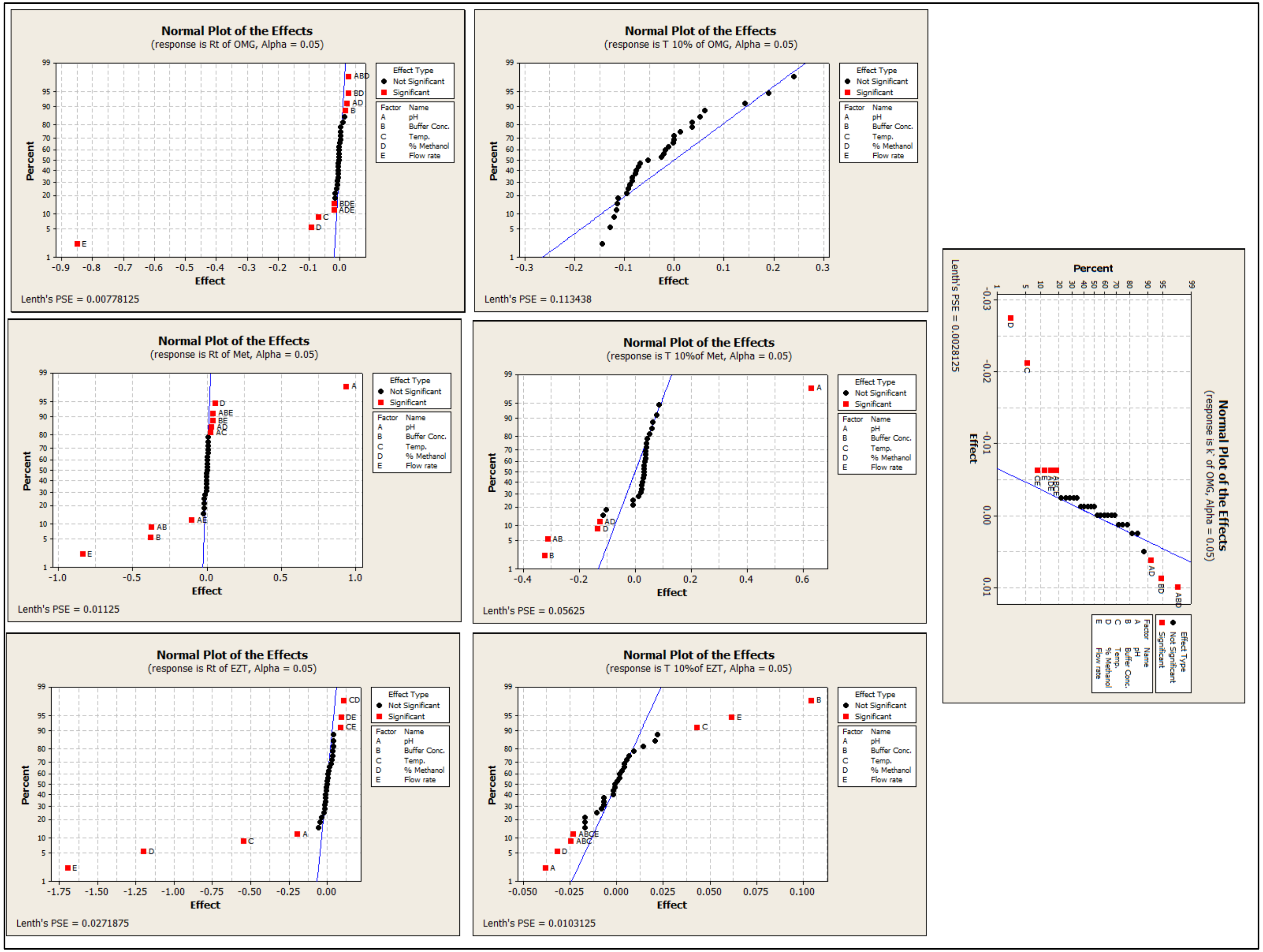

Fig. S4 2^5 FFD normal plots of the effects on the chromatographic responses at alpha = 0.05.

Table S1: 2^5 experimental factorial designs and their dependent responses for RP-HPLC separation of OMG/MET/EZT mixture

Run Order	Center Pt	Blocks	pH	Buffer Conc.	Temp.	% Methanol	Flow rate	k` of OMG	Rt of OMG	width 50% of OMG	T 10% of OMG	Rs of OMG	N of OMG	k` of MET	Rt of MET	width 50% of MET	T 10% of MET	Rs of MET	N of MET	K`of EZT	Rt of EZT	width 50% of EZT	T 10% of EZT	N of EZT
1	1	1	7	0.01	40	67	0.8	0.2	4.33	0.183	1.39	0	1520	0.2	4.33	0.183	1.39	10.03	3108	1.26	8.15	0.267	1.31	5172
2	1	1	6	0.005	40	65	0.8	0.21	4.36	0.149	1.01	13.51	4764	0.02	3.68	0.13	1.45	2.87	4473	1.7	9.71	0.318	1.28	5152
3	1	1	6	0.005	45	67	1	0.15	3.33	0.115	1.14	10.11	4640	0.02	2.95	0.107	1.45	2.02	4222	1.15	6.24	0.224	1.35	4292
4	1	1	7	0.01	40	65	0.8	0.19	4.3	0.187	1.62	0	1643	0.19	4.3	0.187	1.99	12	2934	1.65	9.52	0.327	1.3	4701
5	1	1	7	0.01	40	67	1	0.2	3.47	0.149	1.39	0	1346	0.2	3.47	0.149	1.39	9.63	3014	1.26	6.55	0.227	1.36	4596
6	1	1	7	0.005	45	65	1	0.19	3.44	0.131	1.08	2.25	3796	0.42	4.1	0.219	2.35	7.08	1949	1.43	7.06	0.273	1.38	3698
7	1	1	6	0.01	40	65	0.8	0.22	4.38	0.155	1.1	13.54	4457	0.02	3.67	0.141	1.46	2.84	3791	1.72	9.78	0.315	1.4	5328
8	1	1	6	0.01	40	67	1	0.18	3.41	0.117	1.17	11.45	4705	0.02	2.96	0.112	1.48	2.33	3864	1.31	6.7	0.222	1.39	5038
9	1	1	7	0.01	45	65	1	0.18	3.43	0.152	1.98	0	1759	0.18	3.43	0.152	1.98	10.84	2806	1.49	7.2	0.259	1.43	4297
10	1	1	7	0.01	45	67	0.8	0.2	4.32	0.191	1.44	0	1269	0.2	4.32	0.191	1.44	9.15	2861	1.15	7.72	0.248	1.36	5386
11	1	1	7	0.01	40	65	1	0.21	3.52	0.132	1.07	0	1420	0.18	3.42	0.18	2.1	11.33	2014	1.66	7.7	0.265	1.4	4664
12	1	1	7	0.01	45	65	0.8	0.19	4.27	0.171	2.04	0	1365	0.19	4.27	0.171	2.04	11.01	3480	1.4	8.64	0.297	1.37	4682
13	1	1	7	0.005	45	67	1	0.15	3.34	0.124	1.06	2.83	4048	0.45	4.19	0.232	2.35	4.88	1789	1.1	6.1	0.229	1.34	3843
14	1	1	6	0.005	40	65	1	0.21	3.5	0.126	1.07	12.83	4262	0.02	2.95	0.11	1.47	2.73	4021	1.7	7.82	0.271	1.31	4625
15	1	1	7	0.005	40	65	0.8	0.22	4.4	0.152	1.01	1.81	4603	0.4	5.03	0.255	2.52	9.49	2095	1.66	9.57	0.31	1.22	5208
16	1	1	6	0.005	45	67	0.8	0.16	4.16	0.134	1.13	10.87	5366	0.03	3.69	0.131	1.5	2.13	4410	1.16	7.77	0.258	1.23	5030
17	1	1	6	0.01	40	65	1	0.21	3.51	0.13	1.12	12.76	4044	0.02	2.94	0.116	1.46	2.7	3580	1.7	7.84	0.271	1.41	4643
18	1	1	6	0.005	45	65	1	0.18	3.43	0.128	1.01	11.05	3964	0.02	2.95	0.109	1.49	2.38	4051	1.49	7.23	0.277	1.29	3777
19	1	1	7	0.005	45	65	0.8	0.19	4.29	0.156	1.05	2.21	4191	0.42	5.1	0.277	2.53	7.48	1887	1.44	8.8	0.306	1.31	4581
20	1	1	7	0.005	40	65	1	0.22	3.53	0.128	1.03	1.8	4262	0.39	4.04	0.203	2.39	9.2	2191	1.65	7.69	0.265	1.28	4656
21	1	1	6	0.01	45	65	1	0.19	3.46	0.127	1.11	11.74	4111	0.01	2.93	0.116	1.51	2.56	3573	1.52	7.31	0.261	1.52	4368
22	1	1	6	0.005	45	65	0.8	0.2	4.3	0.152	1.05	12.47	4469	0.02	3.66	0.132	1.51	2.65	4243	1.51	9.05	0.297	1.31	4902
23	1	1	7	0.005	40	67	1	0.17	3.407	0.12	1.02	2.64	4474	0.43	4.13	0.205	2.26	6.54	2247	1.25	6.25	0.225	1.2	4634
24	1	1	7	0.005	45	67	0.8	0.16	4.16	0.15	1.01	2.95	4271	0.46	5.27	0.294	2.48	4.9	1774	1.11	7.58	0.264	1.21	4587
25	1	1	6	0.01	40	67	0.8	0.18	4.24	0.144	1.1	11.58	4801	0.03	3.71	0.138	1.45	2.22	4002	1.31	8.32	0.272	1.34	5198
26	1	1	6	0.005	40	67	1	0.18	3.41	0.114	1.15	11.58	4974	0.02	2.96	0.109	1.51	2.35	4138	1.31	6.71	0.222	1.34	5044

27	1	1	6	0.01	45	67	1	0.16	3.36	0.115	1.21	10.34	4723	0.02	2.95	0.112	1.46	2.14	3810	1.17	6.29	0.22	1.46	4554
28	1	1	6	0.01	45	65	0.8	0.2	4.31	0.153	1.11	12.34	4375	0.02	3.65	0.14	1.5	2.64	3791	1.52	9.08	0.302	1.39	4992
29	1	1	6	0.01	45	67	0.8	0.16	4.18	0.145	1.1	10.4	4615	0.03	3.69	0.139	1.46	2.03	3939	1.17	7.8	0.267	1.36	4747
30	1	1	7	0.01	45	67	1	0.16	3.35	0.077	0.76	0.84	1041	0.21	3.5	0.134	1.98	8.78	3776	1.13	6.16	0.224	1.38	4204
31	1	1	7	0.005	40	67	0.8	0.18	4.25	0.145	0.98	2.59	4745	0.43	5.16	0.266	2.53	6.66	2090	1.27	8.16	0.267	1.19	5191
32	1	1	6	0.005	40	67	0.8	0.18	4.23	0.142	1.06	11.72	4893	0.03	3.72	0.133	1.49	2.19	4348	1.31	8.31	0.268	1.27	5327

Table S2: System suitability parameters for the RP-HPLC determination of OMG/MET/EZT mixture

Parameter	OMG		MET		EZT	
	Factorial design value	Mean ± SD[a], RSD[b]%	Factorial design value	Mean ± SD, RSD%	Factorial design value	Mean ± SD, RSD%
**k'**	0.17	0.15 ± 0.07, 0.07%	-	-	-	-
**Rt (min)**	4.15	4.12 ± 0.04, 0.04%	4.89	4.92 ± 0.035, 0.035%	7.51	7.49 ± 0.042, 0.042%
**$T_{10\%}$**	1.13	1.31 ± 0.06, 0.06%	2.15	2.56 ± 0.058, 0.058%	1.26	1.38 ± 0.08, 0.08%

[a] Standard deviation (three replicates) (Practical estimation).
[b] Percentage relative standard deviation (three replicates).

Table S3: Accuracy and assay results for the determination of OMG, MET, and EZT in laboratory prepared mixtures by the proposed method

Parameter	Conc. taken (µg/mL)			% recovery [a]		
	OMG	MET	EZT	OMG	MET	EZT
**Mix.1**	0.25	5.0	0.1	102.48	100.24	98.50
**Mix.2**	0.5	10.0	0.2	100.66	98.39	99.35
**Mix.3**	0.75	15.0	0.3	97.59	100.17	101.63
**Mix.4**	1.0	20.0	0.4	100.11	101.92	99.83
**Mix.5**	1.25	25.0	0.5	100.58	98.96	99.64
**Mean ± SD**				100.28±1.76	99.94±1.36	99.79±1.15
**% RSD**				1.75	1.36	1.15

[a] Mean of three separate determinations.

Table S4: Intra-day and inter-day precision data for the determination of the studied drugs by the proposed method

Analyte	Conc. taken (µg/mL)	Intra-day [a]			Inter-day [b]		
		Conc. found ± S.D. (µg/mL)	% RSD	%Error [c]	Conc. found ± S.D. (µg/mL)	% RSD	%Error [c]
OMG	**0.5**	0.51 ± 1.47	1.45	0.84	0.49 ± 0.61	0.61	0.35
	**0.75**	0.75 ± 0.62	0.61	0.35	0.74 ± 1.43	1.44	0.83
	**1.0**	0.99 ± 0.66	0.67	0.39	0.99 ± 0.34	0.34	0.2
MET	**10.0**	9.97 ± 0.9	0.9	0.52	9.95 ± 0.99	1.0	0.57
	**15.0**	15.17 ± 0.25	0.25	0.14	15.09 ± 0.47	0.47	0.27
	**20.0**	19.93 ± 0.51	0.52	0.3	19.95 ± 0.61	0.61	0.35
EZT	**0.2**	0.21 ± 1.54	1.5	0.87	0.21 ± 1.32	1.28	0.74
	**0.3**	0.302 ± 0.92	0.92	0.53	0.3 ± 1.04	1.04	0.6
	**0.4**	0.39 ± 0.86	0.86	0.5	0.39 ± 1.12	1.12	0.65

Each result is the average of three separate determinations.

[a] Within the day

[b] Three consecutive days

[c] % Error= % RSD/ $\sqrt{n}$

Table S5: Robustness evaluation of the proposed method

Drug	OMG					MET				EZT			
**% Methanol (67.0% ± 1%)**													
**Variation**	**% Recovery**	**% RSD**	**Rt**	**K`**	**$T_{10\%}$**	**% Recovery**	**% RSD**	**Rt**	**$T_{10\%}$**	**% Recovery**	**% RSD**	**Rt**	**$T_{10\%}$**
**66.0 %**	98.97	1.24	4.19	0.16	1.32	99.36	1.16	5.06	2.54	97.76	1.65	7.83	1.37
**67.0 %**	100.48	1.29	4.16	0.16	1.31	100.34	1.2	5.12	2.56	99.54	1.51	7.58	1.38
**68.0 %**	97.93	1.31	4.11	0.15	1.31	101.94	1.43	5.09	2.53	99.38	1.72	7.37	1.38
**Flow rate (0.814 ± 0.1 mL/min)**													
**0.714**	101.11	1.09	4.38	0.14	1.3	99.58	1.13	5.38	2.58	98.36	1.38	8.24	1.38
**0.814**	100.35	1.06	4.16	0.16	1.31	100.93	1.25	5.12	2.56	99.89	1.19	7.58	1.38
**0.914**	102.46	1.07	3.98	0.2	1.32	100.32	1.37	4.57	2.56	97.24	1.43	7.17	1.36
**Buffer strength (6.6 mM±0.1)**													
**6.5 mM**	100.89	1.13	4.11	0.15	1.33	99.87	1.06	5.09	2.57	100.64	1.14	7.6	1.37
**6.6 mM**	100.12	1.07	4.16	0.16	1.31	99.95	1.2	5.12	2.56	100.26	1.03	7.58	1.38
**6.7 mM**	100.41	1.11	4.2	0.16	1.3	98.76	1.13	5.14	2.54	99.87	1.16	7.57	1.36
**Temperature (45°C ± 1°C)**													
**44°C**	97.21	1.59	4.13	0.16	1.32	98.43	1.29	5.13	2.52	98.37	1.54	7.57	1.35
**45°C**	100.41	1.68	4.16	0.16	1.31	99.69	1.15	5.12	2.56	100.16	1.04	7.58	1.38
**46°C**	98.06	1.62	4.19	0.16	1.31	97.84	1.39	5.1	2.55	101.09	1.63	7.59	1.39
**pH (7 ± 0.1)**													
**6.9**	100.05	0.54	4.15	0.16	1.3	99.85	0.97	5.1	2.55	97.96	1.46	7.57	1.37
**7**	1100.88	0.41	4.16	0.16	1.31	100.52	0.89	5.12	2.56	99.87	0.99	7.58	1.38
**7.1**	100.44	0.63	4.18	0.16	1.3	100.32	1.06	5.14	2.53	98.26	1.38	7.6	1.37

Table S6: Application of the proposed method for the determination of OMG, MET and EZT in spiked human plasma

Drug	OMG		MET		EZT	
**Parameter**	**Conc. taken (µg/mL)**	**%Found [a]**	**Conc. taken (µg/mL)**	**%Found [a]**	**Conc. taken (µg/mL)**	**%Found [a]**
	0.20	95.50	1.0	94.32	0.10	94.3
	0.225	102.80	2.5	100.55	0.20	105.7
	0.25	103.44	4.0	104.79	0.30	98.1
	0.30	98.07	4.5	100.06		
			5.0	96.97		
Mean ± SD		99.95±3.81		99.34±3.95		99.37±5.81

[a] Mean of three separate determinations.

Table S7: Assay results for the determination of three mixtures of the studied drugs in spiked human plasma by the proposed method

Parameter	Conc. taken (µg/mL)			Percentage recovery [a]		
	OMG	MET	EZT	OMG	MET	EZT
**Mix.1**	0.20	4.0	0.10	101.95	101.76	94.4
**Mix.2**	0.225	4.5	0.20	96.53	96.87	105.55
**Mix.3**	0.25	5.0	0.30	101.56	101.41	98.13
**Mean ± SD**				100.01±3.02	100.01±2.73	99.36±5.68
**% RSD**				3.02	2.73	5.71

[a] Mean of three separate determinations.
